# Prevalence of Anemia in Patients With Rheumatoid Arthritis Presenting at Multi-organization Tertiary Care Hospitals

**DOI:** 10.7759/cureus.72418

**Published:** 2024-10-26

**Authors:** Jamal Shah, Abubakar Farooq, Saeedullah Zadran, Zia H Kakar, Muhammad Zarrar, Hafiz Muhammad Hamza Shafique Bhatti

**Affiliations:** 1 General Internal Medicine, Khyber Teaching Hospital, Peshawar, PAK; 2 Research and Development, ClinRe, Arlington, USA; 3 Medicine, Fatima Memorial Hospital, Lahore, PAK; 4 Internal Medicine, Nishtar University and Hospital, Multan, PAK; 5 Rheumatology/General Medicine, Pakistan Institute of Medical Sciences (PIMS), Islamabad, PAK; 6 Medicine, Peoples University of Medical and Health Sciences for Women (PUMHSW), Nawabshah, PAK; 7 Medicine, Lahore General Hospital, Lahore, PAK; 8 Medicine/Surgery, Chengde Medical University, Chengde, CHN

**Keywords:** anemia, c-reactive protein, disease activity score, erythrocyte sedimentation rate, inflammation, rheumatoid arthritis

## Abstract

Background: Rheumatoid arthritis (RA) patients often have anemia, which is frequently made worse by the severity of the illness and ongoing inflammation.

Objective: The objective of this study is to determine the prevalence of anemia among patients with RA presenting at a tertiary care hospital and to explore the relationship between anemia and various clinical parameters of RA.

Methodology: This cross-sectional research evaluated anemia in 330 RA patients in three different hospitals in Pakistan between January and December of 2023. Ages 18 and above, RA diagnosis as defined by the American College of Rheumatology, and permission were the inclusion criteria. Incomplete data or secondary anemic causes were the exclusion criteria. Medical records and interviews were used to gather information on demographics, hemoglobin levels, inflammatory markers, and the severity of RA. SPSS was utilized for the statistical analysis, with significance set at p < 0.05 and Chi-square testing employed to look for relationships between anemia.

Results: In this study of 330 RA patients, anemia was present in 180 patients (54.55%). The mean hemoglobin level among these patients was 11.41 ± 1.87 g/dL, and the red blood cell count averaged 4.26 ± 0.69 million/µL. Anemic patients had a higher Disease Activity Score (DAS28) with a mean of 5.23 ± 1.42, compared to 4.98 ± 1.35 in the 150 non-anemic patients (45.45%). Elevated C-reactive protein (CRP) levels were observed in 150 (83.33%) of the anemic patients, with a mean CRP of 28.79 ± 12.56 mg/L, while 160 (88.89%) of the anemic patients had an elevated erythrocyte sedimentation rate (ESR), with a mean ESR of 45.17 ± 16.29 mm/hr. Significant associations were found between anemia and longer disease duration (p = 0.042), higher RA severity (p = 0.002), and increased inflammatory markers (p < 0.001).

Conclusion: Anemia is prevalent among RA patients and is significantly associated with higher disease severity, longer disease duration, and elevated inflammatory markers.

## Introduction

A common hematological disorder called anemia is defined by a lack of hemoglobin or red blood cells, which reduces the blood's ability to deliver oxygen [1,2]. Many chronic disorders coexist with this syndrome, with rheumatoid arthritis (RA) being the most significant [3]. An extensive range of related problems, including systemic inflammation, are brought on by RA, a complicated inflammatory illness that predominantly affects the synovial joints [4]. The complex relationship between anemia and RA makes diagnosis and treatment very difficult, highlighting the need for more research on this comorbidity [5].

The process of making red blood cells, known as erythropoiesis, may be significantly impacted by chronic inflammation, which is a defining feature of RA [6]. Anemia may arise in people with RA due to systemic inflammation in a number of ways. For example, it is known that inflammatory cytokines like interleukin-6 (IL-6) and tumor necrosis factor-alpha (TNF-alpha) may interfere with iron metabolism and erythropoiesis, leading to anemia of inflammation (AI) or anemia of chronic disease (ACD) [7]. Further confounding the anemic picture in these patients is the use of biologics and disease-modifying anti-rheumatic medications (DMARDs), which, while beneficial in treating RA symptoms, may also have an influence on hematologic markers [8].

Anemia prevalence in individuals with RA may vary greatly depending on a number of variables, including the severity and duration of the illness as well as the existence of concomitant comorbidities [9]. According to recent research, a significant fraction of RA patients have anemia, which may worsen the disease's overall burden and impair quality of life and physical performance [10,11]. Due in part to the similarities in symptoms between RA and anemia as well as the variations in diagnostic and therapeutic modalities, anemia in RA is still underdiagnosed and undertreated despite being frequent [12].

Thus, it is crucial to comprehend the prevalence and features of anemia in RA patients in order to enhance patient treatment and results. Thorough evaluations may result in improved care plans that are customized to each RA patient's requirements, thereby improving their quality of life.

Research objective

The research objective is to determine the prevalence of anemia among patients diagnosed with RA across three tertiary care hospitals in Pakistan and to examine the associations between anemia and various clinical characteristics of RA, including disease severity, treatment regimens, and inflammatory markers.

## Materials and methods

Study design and setting

This cross-sectional observational study was conducted from January to December 2023 at Khyber Teaching Hospital Peshawar, Lahore General Hospital, and Nishtar University Hospital Multan. The primary aim of the study was to investigate the prevalence of anemia in patients diagnosed with RA and to assess the association between anemia and various clinical characteristics of RA. We adhered to the American College of Rheumatology (ACR) criteria for diagnosing RA and collected comprehensive clinical, demographic, and lifestyle data from all participants. Additionally, data on lifestyle factors such as smoking status, alcohol consumption, and physical activity were collected to explore potential influences on anemia risk. Furthermore, genetic predispositions for both RA and anemia were recorded where applicable, as well as treatment regimens including the use of disease-modifying antirheumatic drugs (DMARDs) and biologics.

Inclusion and exclusion criteria

Patients included in the study were diagnosed with RA according to the ACR criteria, which consider factors such as joint involvement, serology (rheumatoid factor and anti-citrullinated protein antibody), acute-phase reactants (C-reactive protein and erythrocyte sedimentation rate), and duration of symptoms (at least six weeks). Participants were required to be at least 18 years of age and provide informed consent. To better account for age-related factors, we stratified participants into relevant age groups (e.g., 18-30, 31-40, 41-50, 51-60, and above 60). The study also included information on the patients' disease duration to assess the long-term impact of RA on anemia risk. Patients with secondary causes of anemia unrelated to RA, such as gastrointestinal bleeding, cancer, or other chronic diseases, were excluded from the study, as were those with incomplete medical records or those who refused participation.

Sample size

A consecutive sampling method was employed to recruit participants, ensuring that all eligible patients presenting to the hospitals during the study period were included. This sampling method aimed to minimize bias and increase the representativeness of the sample. Using the World Health Organization (WHO) algorithm for cross-sectional studies, the sample size was calculated as 330, which would provide a statistically significant estimate of anemia prevalence and its association with clinical characteristics in RA patients.

Data collection

Data were collected through a combination of patient interviews and medical record reviews. Demographic data included patient age, gender, and body mass index (BMI). Lifestyle factors, including smoking status, alcohol consumption, and physical activity levels, were also recorded, as these factors may impact both RA severity and anemia risk. Clinical data were gathered on RA-specific characteristics, including disease duration, severity (based on the Disease Activity Score 28, or DAS28), and inflammatory markers such as C-reactive protein (CRP) and erythrocyte sedimentation rate (ESR). Genetic predisposition to RA and anemia was documented where available, to explore potential hereditary influences. Hematological data, including red blood cell counts and hemoglobin levels, were also recorded to assess the presence and severity of anemia.

Treatment regimens

Comprehensive documentation of treatment regimens was conducted, focusing on FDA-approved minimum doses and durations. The study included methotrexate, which was administered at a minimum dose of 7.5 mg/week for at least three months, hydroxychloroquine at a minimum dose of 200 mg/day for a duration of at least six months, and sulfasalazine, starting with an initial dose of 500 mg/day and increasing to 1 g/day after two weeks, maintained for a minimum of three months. Biologics such as adalimumab were given at an initial dose of 40 mg subcutaneously every other week after an 80 mg loading dose, while etanercept was prescribed at a minimum of 50 mg subcutaneously once weekly for long-term use, and infliximab was initiated at 3 mg/kg at weeks 0, 2, and 6, followed by doses every eight weeks. Corticosteroids, particularly prednisone, started at a minimum of 10 mg/day, with a standard treatment duration not exceeding three months unless specified otherwise by a rheumatologist. This detailed account of treatment regimens allowed for a more nuanced analysis of their impact on anemia status.

Statistical analysis

Descriptive statistics were used to summarize the demographic and clinical characteristics of the study population, with continuous variables such as age, hemoglobin levels, and CRP presented as means with standard deviations (SD), and categorical variables like gender and disease severity as frequencies and percentages. To compare differences between anemic and non-anemic patients, independent t-tests assessed differences in normally distributed continuous variables (e.g., disease duration and hemoglobin levels), while Mann-Whitney U tests were used for non-normally distributed variables (e.g., CRP and ESR). Chi-square tests compared categorical variables such as gender, disease severity (DAS28), anemia prevalence, and treatment regimens (including DMARDs, biologics, and corticosteroids). Weighted multivariate logistic regression was employed to assess the association between anemia and RA-related clinical features, controlling for confounders like age, sex, smoking status, and medication use (including DMARDs, biologics, and corticosteroids), generating odds ratios (OR) with 95% confidence intervals (CI). Spearman correlation analysis was used to examine the relationship between inflammatory markers (CRP, ESR), dietary inflammatory index (DII), healthy eating index (HEI), and anemia due to non-normal variable distributions. Subgroup analyses stratified the sample by demographic factors such as age, gender, race, smoking, and drinking status, while sensitivity analyses adjusted for additional confounders like antirheumatic medication, family poverty income ratio (PIR), and CRP to ensure robustness. Stepwise regression identified the most significant predictors of anemia, and a nomogram model was developed to estimate individual anemia risk, with its discriminatory ability validated via the receiver operating characteristic (ROC) curve, quantified by the area under the curve (AUC). All effect estimates included 95% CIs, and statistical significance was set at p-values less than 0.05, with analyses conducted using IBM SPSS Statistics for Windows, Version 25 (Released 2017; IBM Corp., Armonk, New York, United States).

Ethical approval

Ethical approval for this study was obtained from the Institutional Review Board (IRB) of MTI Khyber Teaching Hospital Peshawar (561/DME/KTH). All patients provided written informed consent before participation, and all procedures adhered to the ethical guidelines for human research.

## Results

The 330 research participants with RA were grouped according to their hematological and demographic parameters as shown in Table 1. With a mean age of 55.21 ± 10.67 years, the age distribution included 25 patients (7.58%) aged 18-30, 55 patients (16.67%) aged 31-40, 115 patients (34.85%) aged 41-50, 90 patients (27.27%) aged 51-60, and 45 patients (13.64%) aged beyond 60. There were 210 females (63.64%) and 120 men (36.36%) in the gender distribution. Red blood cell counts average 4.26 ± 0.69 million/µL, whereas hemoglobin levels average 11.41 ± 1.87 g/dL. The average body mass index (BMI) was 4.12 kg/m^2^, or 24.58 ± 1.87. It was found that 180 patients (54.55%) had anemia, and 150 patients (45.45%) did not (non-anemia). 

**Table 1:** Demographic and Hematological Characteristics of Study Participants RA: Rheumatoid arthritis

Characteristic		Number of Patients; N (%)
Age Groups (Years)	18-30	25 (7.58)
	31-40	55 (16.67)
	41-50	115 (34.85)
	51-60	90 (27.27)
	Above 60	45 (13.64)
	Mean ± SD	55.21 ± 10.67
Gender	Male	120 (36.36)
	Female	210 (63.64)
Hemoglobin Levels (g/dL)	Mean ± SD	11.41 ± 1.87
Red Blood Cell Count (million/µL)	Mean ± SD	4.26 ± 0.69
Body Mass Index (BMI) (kg/m²)	Mean ± SD	24.58 ± 4.12
Lifestyle Factors	Current Smokers	60 (18.18)
	Regular Drinkers	90 (27.27)
	Regular Physical Activity	150 (45.45)
Genetic Predisposition	Family History of RA	75 (22.73)
	Family History of Anemia	50 (15.15)
Distribution of Anemia Among RA Patients	Anemia	180 patients (54.55%)
	Non-anemia	150 patients (45.45%)

**Figure 1:**
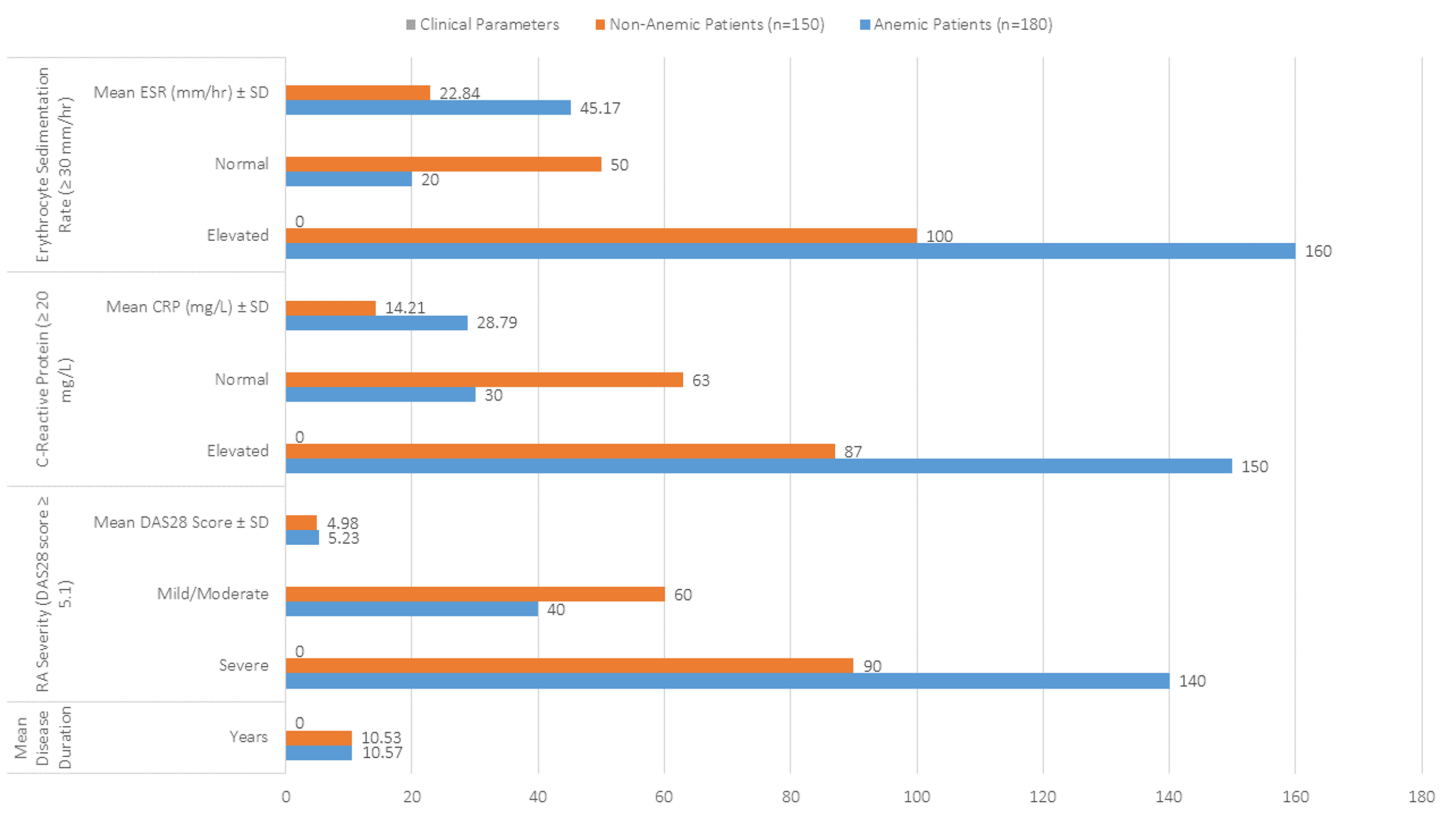
Comparison of Participants by Anemia Status

The comparison of clinical parameters between anemic and non-anemic RA patients reveals significant differences in disease severity and inflammatory markers. While the mean disease duration was similar for both groups (10.57 years in anemic patients and 10.53 years in non-anemic), anemic patients exhibited greater RA severity, with 77.78% classified as severe compared to 60.00% of non-anemic patients as shown in Figure 1. The mean DAS28 score was also higher in the anemic group (5.23 vs. 4.98). Inflammatory markers were notably elevated in anemic patients, with 83.33% having high CRP levels (≥ 20 mg/L) compared to 58.00% of non-anemic patients, and mean CRP levels were significantly higher in anemic individuals (28.79 vs. 14.21 mg/L). Similarly, ESR was elevated in 88.89% of anemic patients, with a mean of 45.17 mm/hr, compared to 66.67% and 22.84 mm/hr in non-anemic patients. These findings suggest that anemia in RA patients is closely associated with increased disease severity and higher levels of systemic inflammation.

Anemia in RA patients was significantly associated with several clinical and treatment-related factors. Patients with anemia were more likely to have a disease duration of 10 years or more (66.67% vs. 53.33%, p = 0.042), suggesting that prolonged RA may increase the risk of anemia (Table 2). The mean duration was similar between both groups (10.57 ± 8.25 years for anemic patients vs. 10.53 ± 8.12 years for non-anemic patients, p = 0.978), indicating that while long disease duration is related to anemia, its severity might not differ based on the exact length of illness. RA severity was notably higher in anemic patients, with 77.78% having severe disease (DAS28 ≥ 5.1) compared to 60.00% in the non-anemic group (p = 0.002), and the mean DAS28 score was also significantly higher in the anemic group (5.23 ± 1.42 vs. 4.98 ± 1.35, p = 0.024). This indicates that more severe RA is strongly linked to anemia. Inflammatory markers, including CRP and ESR, were elevated in the anemic group. Anemic patients had a significantly higher mean CRP level (28.79 ± 12.56 mg/L vs. 14.21 ± 8.35 mg/L, p < 0.001), and 83.33% had elevated CRP (≥ 20 mg/L) compared to 58.00% of non-anemic patients (p < 0.001). Similarly, the mean ESR was much higher in anemic patients (45.17 ± 16.29 mm/hr vs. 22.84 ± 12.36 mm/hr, p < 0.001), with 88.89% of anemic patients having elevated ESR (≥ 30 mm/hr) compared to 66.67% in the non-anemic group (p = 0.001), emphasizing the strong link between anemia and inflammation in RA. Treatment regimens also differed between the groups. A significantly higher proportion of anemic patients were on DMARDs (83.33% vs. 60.00%, p < 0.001), particularly methotrexate (61.11% vs. 43.33%, p = 0.003) and sulfasalazine (13.89% vs. 6.67%, p = 0.045). Biologics use was more frequent in anemic patients (55.56% vs. 33.33%, p < 0.001), particularly anti-TNF biologics (36.11% vs. 20.00%, p = 0.002). Corticosteroid use was also significantly higher among anemic patients (44.44% vs. 26.67%, p = 0.001), with low-dose prednisolone (≤ 10 mg/day) being more common (33.33% vs. 20.00%, p = 0.012). These findings suggest that more aggressive RA treatment may be linked to the presence of anemia, either due to the disease's severity or the side effects of these medications.

**Table 2:** Comparison of RA Severity, Inflammation, and Treatment Regimens by Anemia Status The chi-square test was used to compare categorical variables: Disease duration (≥ 10 years), RA severity (DAS28 ≥ 5.1), C-reactive protein (CRP), erythrocyte sedimentation rate (ESR), and treatment regimens (DMARDs, Biologics, Corticosteroid use); an independent t-test was applied for comparing normally distributed continuous variables: Disease Duration (mean years), RA Severity (DAS28 scores), CRP, and ESR (mean values); the Mann-Whitney U test was applied for non-normally distributed variables; and statistical significance is indicated by a p-value less than 0.05. DMARD: Disease-modifying anti-rheumatic medication; RA: rheumatoid arthritis

Variables	Sub-groups	Anemic Patients (n=180)	Non-anemic Patients (n=150)	p-value
Disease Duration (≥ 10 years)	Yes	120 (66.67)	80 (53.33)	0.042*
	No	60 (33.33)	70 (46.67)	
Duration in Years	Mean ± SD	10.57 ± 8.25	10.53 ± 8.12	0.978
RA Severity (DAS28 score ≥ 5.1)	Severe	140 (77.78)	90 (60.00)	0.002*
	Mild/Moderate	40 (22.22)	60 (40.00)	
	Mean ± SD	5.23 ± 1.42	4.98 ± 1.35	0.024*
C-Reactive Protein (≥ 20 mg/L)	Elevated	150 (83.33)	87 (58.00)	<0.001*
	Normal	30 (16.67)	63 (42.00)	
	Mean ± SD	28.79 ± 12.56	14.21 ± 8.35	<0.001*
Erythrocyte Sedimentation Rate (≥ 30 mm/hr)	Elevated	160 (88.89)	100 (66.67)	0.001*
	Normal	20 (11.11)	50 (33.33)	
	Mean ± SD	45.17 ± 16.29	22.84 ± 12.36	<0.001*
Treatment Regimens	DMARD Use	150 (83.33)	90 (60.00)	<0.001*
	Methotrexate Use	110 (61.11)	65 (43.33)	0.003*
	Hydroxychloroquine Use	40 (22.22)	25 (16.67)	0.234
	Sulfasalazine Use	25 (13.89)	10 (6.67)	0.045*
	Biologics Use	100 (55.56)	50 (33.33)	<0.001*
	Anti-TNF Biologics (e.g., Infliximab, Etanercept)	65 (36.11)	30 (20.00)	0.002*
	Other Biologics (e.g., Tocilizumab, Rituximab)	35 (19.44)	20 (13.33)	0.194
	Corticosteroid Use	80 (44.44)	40 (26.67)	0.001*
	Prednisolone (Low Dose ≤ 10 mg/day)	60 (33.33)	30 (20.00)	0.012*
	Prednisolone (High Dose > 10 mg/day)	20 (11.11)	10 (6.67)	0.202

## Discussion

This study's finding of a 54.55% prevalence of anemia in RA patients aligns with the significant burden of anemia observed in previous studies on RA populations [13]. It is essential to note that the prevalence of anemia in RA is well-documented and not unique to this specific cohort or study. Anemia is a frequent and well-recognized comorbidity in RA, driven largely by the inflammatory nature of the disease. Therefore, our findings underscore the importance of recognizing anemia as a prevalent complication in RA, rather than an unexpected finding within this patient population.

There are several types of anemia commonly associated with RA. The most prevalent is anemia of chronic disease (ACD), also referred to as anemia of inflammation, which results from the body’s inflammatory response hindering red blood cell production. The chronic elevation of cytokines, such as interleukin-6 (IL-6) and tumor necrosis factor-alpha (TNF-alpha), plays a key role in disrupting iron metabolism and inhibiting erythropoiesis [7,14]. Another type, though less frequent, is iron deficiency anemia (IDA), which can occur due to gastrointestinal bleeding from long-term use of nonsteroidal anti-inflammatory drugs or from malabsorption issues [15]. Megaloblastic anemia, due to folate or vitamin B12 deficiency, is also sometimes seen in RA patients, particularly those undergoing long-term treatment with certain DMARDs that can affect nutrient absorption [8].

The treatment of anemia in RA involves a comprehensive approach aimed not only at correcting the hemoglobin deficit but also at addressing the underlying inflammation that contributes to anemia. Iron supplementation may be necessary for patients with IDA, though it is typically ineffective in ACD due to disrupted iron metabolism. For ACD, the cornerstone of treatment is controlling RA disease activity through the use of DMARDs, biologics, or Janus kinase (JAK) inhibitors, which help to reduce the inflammatory burden [7]. Targeting specific inflammatory cytokines, such as IL-6 or TNF-alpha, may improve both RA symptoms and associated anemia [16,17]. In certain cases, erythropoiesis-stimulating agents might be considered, particularly for patients with severe anemia or where rapid correction of hemoglobin levels is necessary [18].

Our study further demonstrated that anemia was significantly associated with higher disease severity, as indicated by the higher Disease Activity Score (DAS28) in anemic patients (5.23 ± 1.42) compared to non-anemic patients (4.98 ± 1.35). This supports earlier findings that anemia exacerbates disease outcomes, potentially worsening physical function and quality of life [15,19]. Additionally, patients with a longer RA duration (≥ 10 years) were more likely to be anemic, highlighting the cumulative impact of chronic inflammation on red blood cell production over time [20]. These results are also consistent with research by Marques et al. [21], which highlighted the involvement of chronic inflammation in the development of anemia of chronic disease by reporting a greater incidence of anemia in RA linked with higher levels of inflammatory markers.

These findings emphasize the necessity for routine screening and early management of anemia in RA patients, particularly those with severe or prolonged disease. Addressing anemia effectively can lead to improvements in physical function, fatigue, and overall quality of life. Future research should prioritize exploring optimal anemia treatment strategies tailored to RA patients, including the use of anti-inflammatory therapies and other targeted approaches to correct anemia while managing the underlying disease. Additionally, longitudinal studies will be crucial to further clarify the relationship between RA disease parameters, anemia progression, and treatment outcomes.

Study strengths and limitations

This study offers several notable strengths that contribute to a better understanding of anemia in RA patients. The use of a standardized diagnostic approach based on the ACR criteria ensures accurate identification of RA, strengthening the internal validity of the results. The inclusion of participants from three diverse tertiary hospitals through consecutive sampling minimizes selection bias, making the sample more representative and enhancing the external validity and generalizability of the findings. The comprehensive data collection on clinical and inflammatory markers allows for an in-depth analysis of their potential impact on anemia status in RA patients. There are also important limitations to consider. Longitudinal studies would be crucial to better understand the temporal progression of anemia in RA and how it responds to treatment over time. Although extensive demographic and clinical data were collected, there could be unmeasured confounding factors, such as genetic predispositions or socioeconomic variables, which may influence the association between RA and anemia. Future studies should explore these aspects in more diverse populations to provide a more comprehensive understanding.

## Conclusions

In our study involving 330 patients with RA, we found that anemia was prevalent among a significant portion of participants, with a notable skew toward females. Anemic patients exhibited higher disease severity and demonstrated elevated inflammatory markers, indicating a strong link between anemia and systemic inflammation. These findings underscore the critical importance of routine screening and management of anemia in RA patients, particularly those with severe or prolonged disease, as addressing anemia could enhance overall patient outcomes and quality of life. Future research should focus on elucidating the mechanisms linking RA and anemia, conducting longitudinal studies to better understand these associations over time, and evaluating the effectiveness of anemia treatments in improving patient health.
